# CANEapp: a user-friendly application for automated next generation transcriptomic data analysis

**DOI:** 10.1186/s12864-015-2346-y

**Published:** 2016-01-13

**Authors:** Dmitry Velmeshev, Patrick Lally, Marco Magistri, Mohammad Ali Faghihi

**Affiliations:** Department of Psychiatry, University of Miami Miller School of Medicine, Miami, FL 33136 USA; Department of Biochemistry & Molecular Biology, University of Miami Miller School of Medicine, Miami, FL 33136 USA; Department of Biomedical Engineering, University of Miami, Coral Gables, FL 33146 USA

**Keywords:** RNA sequencing, User-friendly application, Graphical user interface, Automated pipeline, Platform-independent, Differential gene expression, Long noncoding RNAs

## Abstract

**Background:**

Next generation sequencing (NGS) technologies are indispensable for molecular biology research, but data analysis represents the bottleneck in their application. Users need to be familiar with computer terminal commands, the Linux environment, and various software tools and scripts. Analysis workflows have to be optimized and experimentally validated to extract biologically meaningful data. Moreover, as larger datasets are being generated, their analysis requires use of high-performance servers.

**Results:**

To address these needs, we developed CANEapp (application for Comprehensive automated Analysis of Next-generation sequencing Experiments), a unique suite that combines a Graphical User Interface (GUI) and an automated server-side analysis pipeline that is platform-independent, making it suitable for any server architecture. The GUI runs on a PC or Mac and seamlessly connects to the server to provide full GUI control of RNA-sequencing (RNA-seq) project analysis. The server-side analysis pipeline contains a framework that is implemented on a Linux server through completely automated installation of software components and reference files. Analysis with CANEapp is also fully automated and performs differential gene expression analysis and novel noncoding RNA discovery through alternative workflows (Cuffdiff and R packages edgeR and DESeq2). We compared CANEapp to other similar tools, and it significantly improves on previous developments. We experimentally validated CANEapp’s performance by applying it to data derived from different experimental paradigms and confirming the results with quantitative real-time PCR (qRT-PCR). CANEapp adapts to any server architecture by effectively using available resources and thus handles large amounts of data efficiently. CANEapp performance has been experimentally validated on various biological datasets. CANEapp is available free of charge at http://psychiatry.med.miami.edu/research/laboratory-of-translational-rna-genomics/CANE-app.

**Conclusions:**

We believe that CANEapp will serve both biologists with no computational experience and bioinformaticians as a simple, timesaving but accurate and powerful tool to analyze large RNA-seq datasets and will provide foundations for future development of integrated and automated high-throughput genomics data analysis tools. Due to its inherently standardized pipeline and combination of automated analysis and platform-independence, CANEapp is an ideal for large-scale collaborative RNA-seq projects between different institutions and research groups.

## Background

Rapid development of next-generation sequencing technologies has revolutionized fields of genetics and molecular biology [1]. These tools have enabled unbiased and comprehensive insight into novel mutations and changes in transcriptional and epigenetic processes associated both with normal cellular functioning and disease states. Next-generation RNA sequencing allows direct [2] or indirect sequencing of RNA and provides quantitative and qualitative information of all RNA species in a sample [3, 4]. RNA-seq is a powerful technique that can be applied to obtain genome-wide estimates of relative gene, exon or transcript expression; and to discover previously unannotated transcriptional features, such as novel splice junctions and gene isoforms [5], novel gene loci [6] and fused transcripts [7–10]. RNA-seq data analysis consists of a number of consecutive steps, such as raw data preprocessing, short reads alignment, transcript reconstruction, abundance estimation, filtering of low abundance and spurious elements, differential expression testing, annotation of transcriptional elements based on previous annotations and downstream analysis such as coding potential calculation to identify novel noncoding RNAs. Numerous tools, computational and statistical approaches have been developed for these analysis steps, but there has been little agreement in the field on what combination of tools to use for each particular experimental goal [11, 12]. More importantly a user-friendly, streamlined and flexible analysis pipeline combining a plethora of bioinformatics tools and techniques is missing. Some efforts have been directed toward developing an analysis pipeline or a suite of tools that combines various bioinformatics instruments into a RNA-seq analysis toolkit. However, cumbersome installation, lack of user interface, limited automation and inability to use high-performance server architectures are just a few of the most common drawbacks of the currently available pipelines. Moreover, none of the existing software suits have been validated on real biological datasets and with alternative techniques, which leaves their robustness and accuracy untested.

Here, we developed CANEapp (application for Comprehensive automated Analysis of Next-generation sequencing Experiments), an analysis suite consisting of a Java graphical user interface and an automated bioinformatics pipeline (http://psychiatry.med.miami.edu/research/laboratory-of-translational-rna-genomics/CANE-app). CANEapp is a windows-based installation-free, point and click application that can be launched on any PC or Mac. CANEapp is able to operate on a variety of server infrastructures, including Amazon Cloud and high-performance computational clusters. Analysis with CANEapp is fully automated and scales depending on the amount of resources available, thus making it suitable for analysis of large datasets. CANEapp performs differential gene expression analysis and discovery of novel long noncoding RNA with a combination of established analysis tools and alternative workflows, which allows for comprehensive analysis of data in alternative ways in a single run. Additionally, it formats the data into ready-to-view files and provides automated primer design for qRT-PCR validation.

## Implementation

CANEapp’s Graphical User Interface is implemented on Mac or Windows and requires Java version 7 or above. The computational pipeline is implemented on Linux and has been tested on Ubuntu, CentOS, RedHat, Fedora and Amazon Cloud Linux and requires Python version 2.6 or 2.7. The prerequisite libraries for software installation are installed automatically if the user has root access. Otherwise the prerequisites are compiled in a single shell script that is included with the package and needs to be run by the administrator.

## Results

### CANEapp: flexible and user-friendly multiplatform framework for integrated transcriptome analysis

**Fig. 1:**
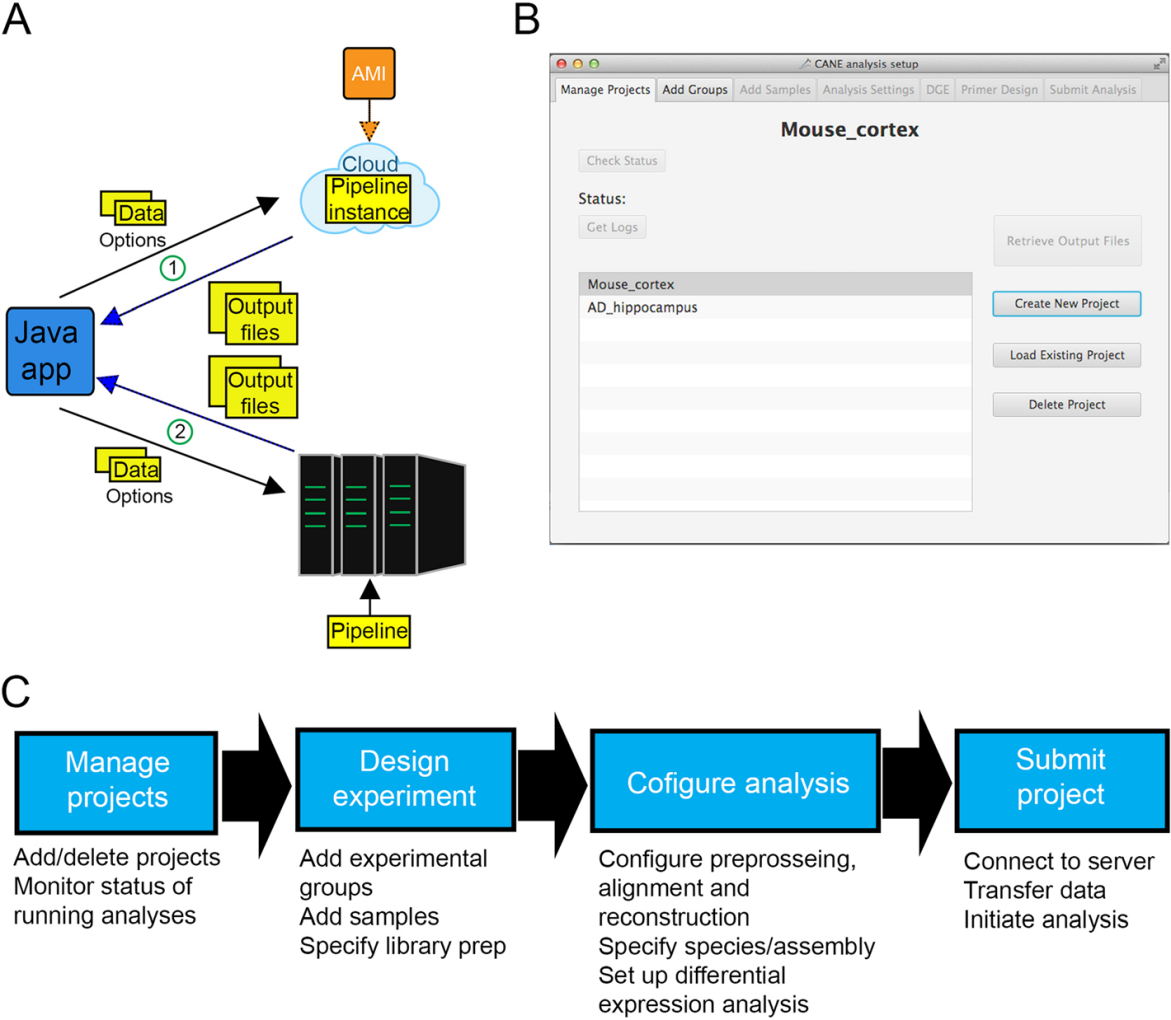
CANEapp and the graphical user interface. **a** General structure of CANEapp. The Java application component is the only user-accessible component and operates on a personal computer to provide a point and click interface to configure RNA-seq analysis. The interface either establishes a connection with an Amazon Cloud instance (1) created using the preconfigured CANEapp Amazon Machine Image (AMI) or with a Unix server, in which case server-side pipeline components are automatically transferred to the server through the GUI. After configuring a project, the GUI communicates with the server side to transfer raw data files and options file and initiate the analysis. **b** Design of the CANEapp’s graphical user interface. **c** CANEapp GUI’s capabilities and project design steps. The Manage Projects tab allows creating, deleting or loading projects from a file. Additionally, user can see the status of the selected project on this tab. The next two tabs allow adding experimental groups and samples. On the Add Samples tab the user can specify the library preparation that has been used before sequencing and define such parameters as single or paired-end sequencing, strand selection and adapter sequences. The Analysis Settings tab is used to set up parameters of separate analysis steps, such as alignment, reconstruction and differential expression analysis. Finally, the last tab is used to specify server address and user credentials and initiate the analysis on the server side

CANEapp is an installation-free analysis framework (Fig. 1a) that allows the user to design, manage and monitor RNA-seq analysis experiments on a personal computer. CANEapp takes advantage of a Java-based graphical user interface to implement our Python-based automated RNA-seq analysis pipeline on a Linux server to perform resource-demanding analysis.

Framework of CANEapp is highly flexible and can be implemented on a variety of server types, including standard Linux servers, Linux servers that use IBM Platform LSF Session Scheduler and Amazon Cloud servers. CANEapp was tested on a number of Linux operation systems, including Ubuntu, CentOS, RedHat Enterprise, Fedora, as well as Amazon Cloud Linux and a CentOS server utilizing IBM Platform LSF Session Scheduler.

The only component that the user needs in order to utilize CANEapp is the GUI (Fig. 1b). The GUI was written in Java with help of NetBeans Integrated Development Environment and JavaFX Scene Builder. Scene Builder was utilized to design most of the GUI’s graphical components, whereas the working scripts were written using NetBeans. Java Secure Channel (JSch) protocols served as the foundation for establishing connection with the server, data upload to the server and data download from the server to the local machine.

The GUI allows easy step-by-step design of RNA-seq analysis projects, set up of analysis configuration, data transfer, project management and status monitoring (Fig. 1c). GUI seamlessly interacts with the server to engage the analysis pipeline that is in essence a “black box”, hidden from the user but containing the components to perform all the required steps of the analysis. The “black box” model insures that user does not have to directly interact with the server or any of the software at any stage of the analysis. This makes CANEapp immediately accessible to any user with little to zero background in bioinformatics or computational science. Moreover, all project configurations are automatically stored in the GUI’s memory, which allows management of running projects on different servers and instant access to project design and settings. Automation saves both computational and hands-on time considerably and removes a requirement of detailed knowledge of computational tools; and together with a point and click interface, CANEapp will allow users without bioinformatics background to perform RNA-seq analysis.

### Automated scalable RNA-seq analysis pipeline for accurate and comprehensive transcriptome analysis

Once the project has been designed and analysis settings have been specified, server address and credentials need to be provided in order to submit the project. The GUI will connect to the server and copy the pipeline components and raw data files together with the project design and settings. After the data transfer is completed, a notification window will appear and analysis will be initiated on the server side through the computational pipeline. Once the analysis is initiated GUI can be closed and reopened at any time to check the status of the particular project.

The analysis pipeline was written in Python and consists of several interacting scripts to perform automated analysis of RNA-seq experiments. The pipeline also generates a status file that is used to communicate with the GUI and keep track of the progress of each project. The GUI evokes the main pipeline script after the raw data files have been transferred to the server. Then the main script guides the construction of analysis framework if it has not been performed before and evokes child processes that perform parallel analysis of samples using other pipeline scripts and software tools. The pipeline automatically passes the analysis settings specified in the GUI to the appropriate pipeline script or software tool. The main script monitors resource usage and completion of analysis of individual samples. After all samples are analyzed, the main script evokes a series of secondary scripts to combine the data and perform filtering and differential gene expression analysis. Finally, the data is formatted into an easy-to-view format and can be downloaded to the local machine through the GUI.

**Fig. 2:**
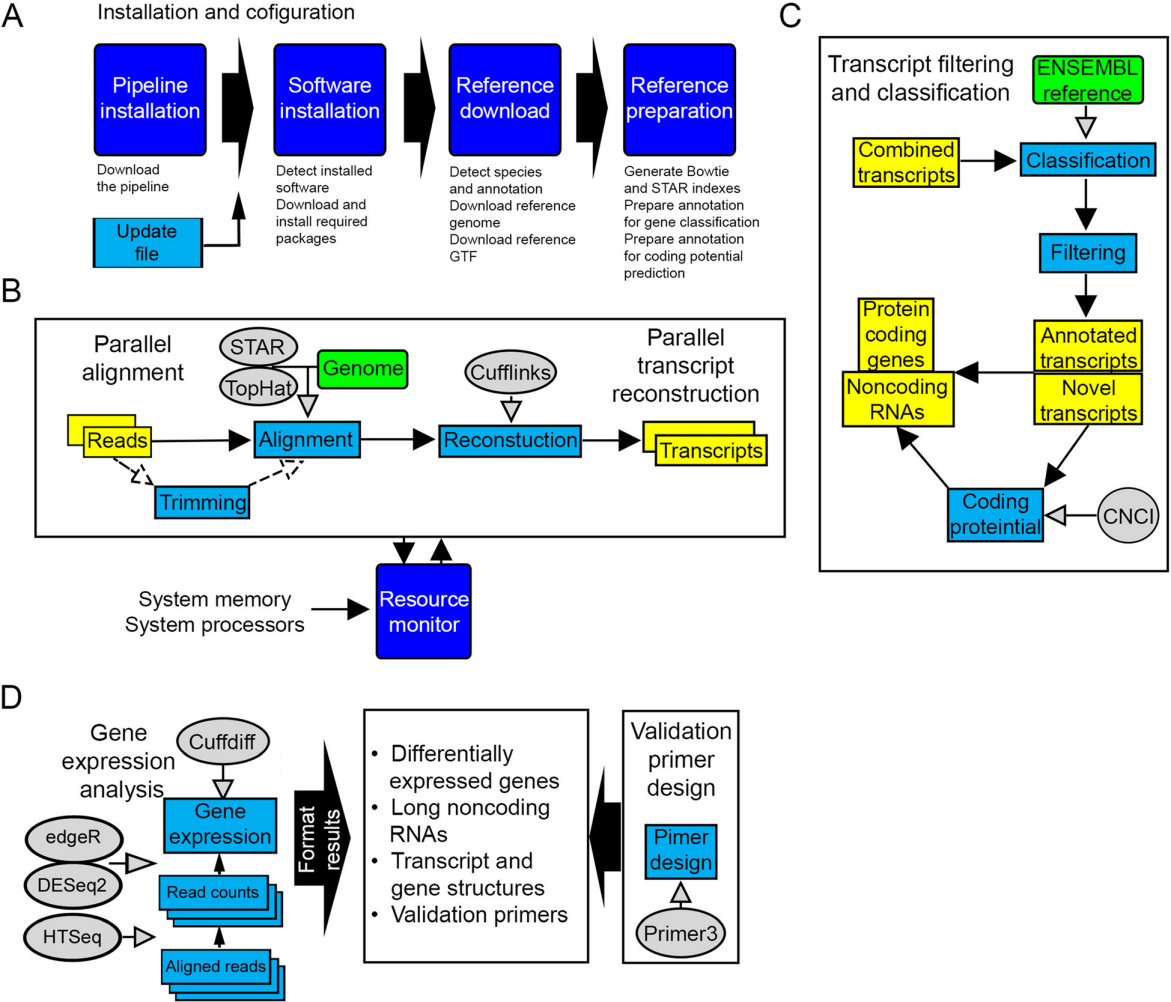
Server-side RNA-seq analysis pipeline. **a** Installation and configuration. First the GUI transfers the pipeline scripts to the server or utilizes pre-installed scripts if Amazon Cloud instance is being used. Then the pipeline detects installed software and downloads and installs all the analysis tools required for the workflow using an update file on our website which is linked to the current version of CANEapp. After that the pipeline downloads required reference files from ENSEMBL. Reference indexes for STAR and TopHat, as well as gene classification files are prepared in the next step. **b** Parallel alignment and reconstruction module. Samples are analyzed in parallel; first the reads go through an optional trimming step and are aligned to the genome with either TopHat or STAR. Aligned reads are used to reconstruct transcripts with Cufflinks. This module includes a resource monitor that optimally distributes available resources between subprocesses. **c** Transcript filtering and classification module. ENSEMBL reference is used to classify genes generated from combining transcript files from all samples. Then the transcripts are filtered to remove potentially spurious single-exon transcripts, and unannotated transcripts and loci are analyzed to predict their ability to code for proteins. **d** Gene expression and results formatting module. Cuffdiff, edgeR and DESeq2 are used to quantify gene expression and identify differentially expression genes. The pipeline converts output files into fully annotated tab-delimited files, as well as GTF files containing differentially expressed genes. The module also contains primer design scripts that automate primer design for qRT-PCR validation of gene expression

The pipeline consists of several modules, the first of which is the installation module (Fig. 2a). This module will download and install all the required software (Table 1), as well as the reference genome and transcriptome files from ENSEMBL, according to the species and assembly specified for the project. The installation module will also build indexes for TopHat [13] and STAR [14] alignment and will prepare the reference annotation for gene classification and coding potential calculation.

**Table 1:** List of software packages and scripts used in CANEapp

Software name	Function	CANE module
SRA tools	FASTQ extraction from the SRA file format	Alignment and reconstruction
TopHat	Read alignment	Alignment and reconstruction
STAR	Read alignment	Alignment and reconstruction
Cufflinks	*Ab initio* transcript reconstruction	Alignment and reconstruction
Cuffcompare	Merging transcripts	Transcript filtering and classification
Samtools	Nucleotide sequence extraction	Transcript filtering and classification
CNCI	Coding potential prediction	Transcript filtering and classification
Cuffdiff	Differential expression testing	Gene expression and results formatting
HTSeq	Counting reads in loci	Gene expression and results formatting
edgeR (R package)	Differential expression testing	Gene expression and results formatting
DESeq2 (R package)	Differential expression testing	Gene expression and results formatting
Primer 3	Primer sequence retrieval	Primer design

The next pipeline module engaged after the installation module is the parallel alignment and reconstruction module (Fig. 2b), which will first perform optional preprocessing of reads. Accepted raw data format is FASTQ or FASTQ files compressed as bz2, tar, gz, tar.gz archives, as well as those saved in the NIH Short Sequence Archive (SRA) format. This step incudes optional extraction of archived files or files in the SRA format and library adapter trimming with our custom Python script in order to remove adapter sequences and improve read alignment and to calculate mean and standard deviation of the insert sizes based on supplied mean and standard deviation of fragment length and library adaptor length. The module will then proceed to perform alignment of RNA-seq reads using TopHat or STAR. TopHat and STAR are used with default parameters, but the user has the ability to specify custom parameters in the GUI when designing the project.

Aligned reads will be further used to perform *ab initio* [15] reconstruction of transcripts using Cufflinks [16], which allows identification of novel, previously unannotated transcriptome features, such as novel long noncoding RNAs. As with TopHat and STAR, the user can specify parameters for Cufflinks in the GUI. Importantly, the alignment and reconstruction module includes a real-time resource monitor that keeps track of the amount of available memory and cores to protect the system from memory or processor overload and ensure optimal resource usage for the fastest performance.

**Fig. 3:**
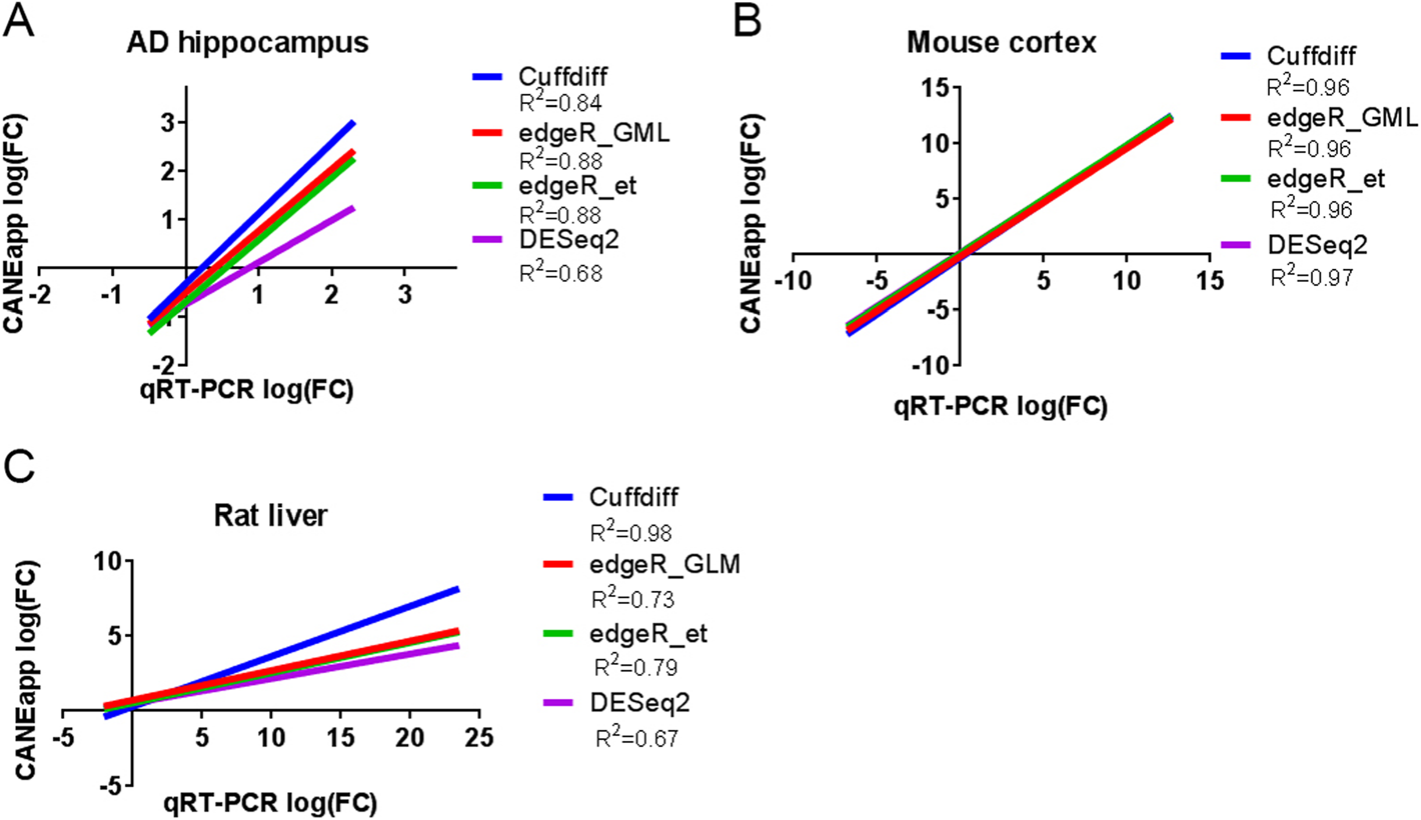
Validation of gene expression changes estimated with CANEapp with quantitative real-time PCR. **a** RNA-seq analysis of hippocampi of Alzheimer’s disease patients and controls. Hippocampal tissue from 4 AD patients and 4 control individuals was used to extract total RNA and perform ribodepletion and strand-specific library preparation. Single-end RNA sequencing was performed on Illumina HiSeq 2000. Fold changes of expression for 2 downregulated and 4 upregulated genes measured with real-time PCR was compared with expression values generated by CANEapp. **b** RNA-seq of developing mouse cortex. Tissue from 4 embryonic day 17 and 3 adult mouse cortical samples was processed to extract polyA-selected RNA and generate paired-end unidirectional sequencing data with Illumina Genome Analyzer IIx. Gene expression estimates of 4 downregulated and 4 upregulated genes were compared between CANEapp and real-time PCR. **c** Fold changes of gene expression for RNA-seq of liver of rats treated with two DNA-damage compounds. The data was produced by paired-end sequencing of polyA-selected RNA on Illumina HiSeq 2000. Fold changes of expression for 2 downregulated and 4 upregulated genes were compared between CANEapp and real-time PCR. R^2^-coefficient of determination

Once all the individual samples have been processed, aligned and reconstructed, the data is passed to the transcript filtering and classification module (Fig. 2c). The module will first combine transcripts from individual samples using Cuffcompare before performing optional transcript filtering described in detail below and in Fig. 3a. Transcript filtering is performed using our custom scripts and in general improves the accuracy of abundance estimation and detection of novel transcripts by removing spurious transcripts such as intronic and pre-mRNA species. The transcripts are then classified into annotated and unannotated transcripts. Annotated transcripts are further assigned a gene biotype according to the ENSEMBL reference, whereas the protein-coding potential of the unannotated transcripts is predicted using Coding-NonCoding Index (CNCI) software [17] and further sub classified into novel noncoding RNAs and potentially novel protein-coding genes.

The final step of the analysis is differential gene expression analysis, which is performed by Cuffdiff [18], or by the R packages edgeR [19] and DESeq2 [20] using HTSeq [21] to count reads prior to processing the data in R. The user can select to either perform analysis with one of the three workflows for differential gene expression analysis (Cufflinks, edgeR and DESeq2) or to run all three of them in parallel. The results of the entire analysis are formatted to create a single tab-delimited file for each differential gene expression analysis method containing expression values, fold changes, statistics and metadata such as gene classification and chromosomal location (Fig. 2d).

The filtered and annotated Gene Transfer File (GTF) as well as aligned reads for individual samples serves as the input for Cuffdiff and HTSeq. Cuffdiff performs differential gene expression analysis and generates normalized abundance estimates, fold changes of gene expression, as well as p and FDR-corrected p values. For analysis with R packages, individual count files generated with HTSeq are combined into one count file, which is supplied to edgeR and DESeq2 together with the parameters defined by the user in the GUI. Cuffdiff, edgeR and DESeq2 output files, together with the GTF files are processed by the pipeline scripts to generate unified data tables containing information on the gene id, name, biotype, read count and abundance (for Cuffdiff) in each sample and group; as well as fold change of expression between groups and p and FDR values. Separate GTF files containing only the differentially expressed genes are also generated. Data tables can be opened in excel to easily interpret the results and rank genes depending on the project goals. GTF files can be displayed in a third-party software such as Integrated Genome Viewer [22] to visualize gene and transcripts structures and genomic locations. The final results files can be easily downloaded through the GUI as soon as the analysis is completed.

After the analysis is finished, qRT-PCR validation primers for specific genes can be designed through the GUI’s Primer Design tab. We automated design of primers for validating sequencing results with qRT-PCR. Our primer design tool searches for a common spliced junction that exists in all isoforms of a gene. In case there are no common junctions the program looks for an exonic region overlapping all the isoforms. After that, Samtools is used to extract the nucleotide sequence of the exons spanning the junction or the exonic region where primers will be designed. Finally, the sequences are supplied to Primer 3 software that designs the primers.

All the intermediate files are stored on the server and can be retrieved by the user through the terminal in case they are required for any downstream applications.

### Comparison of CANEapp to other applications for RNA-seq data analysis

In order to comprehensively compare CANEapp to previously developed and published applications aiming to simplify RNA-seq analysis by providing a graphical user interface, we considered a number of features and contrasted them between the software packages (Table 2). The compared features included: 1- The ability to perform automated analysis of multiple samples and groups through a complete pipeline without the need to perform analysis of each sample at each step of the pipeline. 2- Automated installation of the application and its components without cumbersome command line installation procedures. 3- The possibility to utilize the software on different operation systems and server architectures, including the cloud. 4- The availability of alternative analysis workflows. 5- The ability to efficiently use computational resources and adapt to the amount of data being analyzed to be suitable for analysis of large datasets and efficient allow implementation on high-performance systems. We compared CANEapp to six other published applications for RNA-seq analysis we are aware of. Only free softwares were included in this list.

**Table 2:**
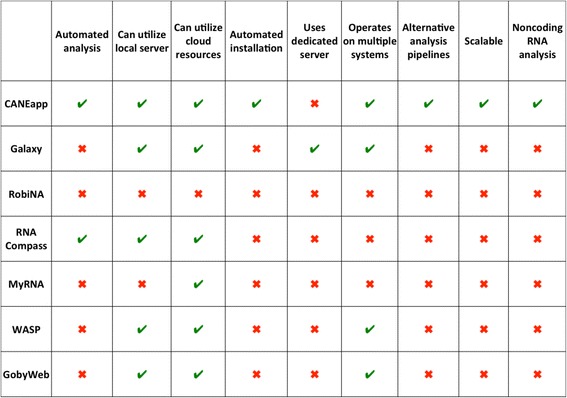
Comparison of CANEapp with previously developed tools for RNA-seq analysis

Note: the green checkmark signifies presence of a feature in a software tool, the red cross means absence of the feature

As can be seen from Table 2, CANEapp possesses all of the abovementioned features, which makes it a powerful, but easy-to-use tool for comprehensive RNA-seq data analysis that can be ported to a variety of server architectures and applied to large datasets without the need for step-by-step analysis or concerns about sufficiency of computational resources (which is handled by the CANEapp’s resource monitor). Although some previously developed and published tools have some of these features, none combine them in one package, which limits their performance and scope of application. For instance, Galaxy offers a number of next-generation sequencing data analysis tools that can be operated through a graphical user interface. However, Galaxy does not offer automation of the analysis, and every step has to be performed manually. Moreover, the scale and speed of analysis through Galaxy server is limited, and if it is to be installed on a local server or cloud it requires installation by a person with computer science skills. Other tools such as RNA Compass offer automation of analysis and work on the cloud in addition to local servers but again, it requires cumbersome installation and lacks other important features highlighted in the Table 2.

Overall, we believe that CANEapp presents significant improvements over previously developed user-friendly applications for RNA-seq analysis and will offer biologists a powerful analysis framework that can be easily ported to their favorite system and used without the need to manually perform any of the installation or analysis steps.

### CANEapp functionality on various Linux server architectures and its performance and accuracy in identifying differentially expressed genes from real datasets

In order to test CANEapp performance and accuracy in estimating gene expression changes in different biological systems and experimental paradigms, we used publically available RNA-seq data from three published studies (Table 3) together with qRT-PCR validation of gene expression changes for several genes for each study. We utilized qRT-PCR data for all the genes validated in each corresponding study; these genes were selected by the authors to either represent a range of fold changes of gene expression or were chosen based on their biological significance. RNA-seq data for testing performance of CANEapp were downloaded from the NIH Short Sequence Archive (SRA) as SRA files and were used directly as input files for CANEapp.

**Table 3:** Description of datasets used to validate CANEapp performance to estimate differential gene expression

Name	Organism	Experimental groups	N of samples	RNA selection protocol	Library preparation	Single or paired-end	GEO
Transcriptomic changes in hippocampi of Alzheimer’s disease patients	Homo sapiens	Alzheimer’s disease vs age- and sex-matched neurologically normal controls	4 vs 4	Ribo-depletion	Illumina directional small RNA prep	single	GSE67333
Transcriptomic changes in embryonic and adult mouse cortex	Mus musculus	E17 cortex vs adult cortex	4 vs 3	Poly-A selection	Illumina mRNA-Seq prep	paired	GSE39866
SEQC Rat liver toxicogenomics study	Rattus norvegicus	N-Nitrosodimethylamine, Aflatoxin B1 vs Vehicle treatments	3 vs 3	Poly-A selection	Illumina TruSeq RNA	paired	GSE55347
			3 vs 4				

The datasets included RNA-seq of hippocampi of Alzheimer’s disease patients and controls (4 AD vs 4 controls) [23], RNA-seq of developing mouse cortex (4 embryonic cortical samples vs 3 adult) [24], and RNA-seq of rat liver from the SEQC Toxicogenomics Study for chemical treatment with two chemical compounds causing DNA damage (N-Nitrosodimethylamine, NIT and Aflatoxin B1, AFL, *N* = 3 for each treatment group, compared to a corresponding control group, *N* = 3 and 4) [25]. The human dataset was generated by sequencing RNA depleted of ribosomal RNAs, whereas mouse and rat RNA-seq data were derived from sequencing of polyA-selected RNA. All three datasets were generated using different library preparation techniques. RNA in human and rat experiments was sequenced on the Illumina HiSeq 2000 machine, whereas mouse RNA-seq data was produced by sequencing RNA on Illumina GA-IIx sequencer. This diversity of experimental paradigms, organisms, RNA preparation, library generation and sequencing techniques allowed us to comprehensively assess the robustness of our analysis tool.

To analyze these datasets, raw data were downloaded from SRA and CANEapp was used to perform analysis on a High-Performance Computing cluster Pegasus2 at the University of Miami and Amazon Elastic Cloud 2 (EC2). In order to comprehensively test the functionality of CANEapp on various Linux architectures, Amazon Machine Images containing distributions of CentOS, Ubuntu and RedHat Linux, as well as Amazon Linux, were used to create instances running these different Linux platforms. All three datasets were analyzed on these instances and the Pegasus2 system using solely the CANEapp application to perform analysis. After completion of the analysis, the generation of functional software binaries from source, as well as reference files, intermediary analysis files and final result files were validated to assure the stability of the pipeline independent of the server architecture.

Raw data were uploaded through CANEapp by selecting the “Upload From Computer” option, and STAR aligner was selected to perform alignment to the latest genome assembly available. For the rest of CANEapp options, the default settings were used. Real-time PCR results for candidate genes from each analyzed dataset were either retrieved from supplementary material for the original publication or received from the authors upon request. Once the analysis was completed, data was downloaded from the server through the GUI, and fold changes in gene expression between experimental groups generated either by Cuffdiff, edgeR using Generalized Linear Model (GLM) or exact test approaches, or DESeq2 were compared with qRT-PCR results for the same gene. For all three datasets, we found perfect correspondence between the direction of gene expression changes estimated from RNA-seq data analyzed with CANEapp using 4 different approaches for differential gene expression analysis and qRT-PCR. All the genes upregulated in RNA-seq were upregulated in qRT-PCR data, and the same was true for downregulated genes (Fig. 3, Table 4). For the human RNA-seq data from hippocampi of Alzheimer’s disease patients and controls, we have compared fold changes in gene expression for 6 genes (4 upregulated and 2 downregulated) (Fig. 3a). The R squared correlation coefficient between the fold changes in RNA-seq and qRT-PCR for these 6 genes is 0.84 for Cuffdiff, 0.88 for both analysis approaches with edgeR (Generalized Linear Model or exact test) and 0.68 for DESeq2; indicating accuracy and robustness of CANEapp performance on real biological RNA-seq data regardless of the analysis approach used. Analysis of gene expression changes in mouse embryonic versus adult cortex with CANEapp and their comparison with qRT-PCR results produced a similar result (Fig. 3b). For the 8 genes validated with qRT-PCR (4 upregulated and 4 downregulated), R squared coefficient between RNA-seq and qRT-PCR data was 0.96 for Cuffdiff and edgeR and 0.97 for DESeq2. In the case of the rat liver toxicology experiment expression of all 8 tested genes (6 upregulated and 2 downregulated) was also successfully validated with qRT-PCR (Fig. 3c). The R squared coefficient between RNA-seq and qRT-PCR data was 0.98 for Cuffdiff, 0.73 for edgeR using GLM, 0.79 for edgeR using exact test and 0.67 for DESeq2. In all three datasets and with all 4 approaches to differential gene expression analysis, correlation of fold changes produced from RNA-seq by CANEapp and qRT-PCR was statistically significant (*p* < 0.05) using two-tailed T test.

**Table 4:** Fold changes of gene expression in three datasets reanalyzed by CANEapp and compared to qRT-PCR results

Gene Name	Cuffdiff	edgeR_GML	edgeR_et	DESeq2	QRT-PCR
Alzheimer’s disease dataset					
SERPINE1	1.41	1.71	1.54	0.98	1.66
TAC1	−1.77	−1.65	−1.85	−1.56	−0.42
ID2	0.98	1.07	0.88	−0.73	1.17
GRM2	0.86	0.98	0.78	0.63	0.44
LINC01314	−0.25	−1.19	−1.38	−1.05	−0.50
RP11-87E22.2	3.63	2.01	1.85	1.31	2.30
Mouse cortex dataset					
Vax1	−2.12	−2.02	−1.74	−1.71	−3.18
Caly	1.93	1.79	2.08	2.09	2.10
Igf2bp1	−9.40	−8.72	−8.45	−8.16	−5.61
Draxin	−5.98	−5.26	−4.98	−4.94	−6.80
Nrp1	−2.17	−2.22	−1.94	−1.92	−2.46
Ttr	11.04	11.18	11.46	11.28	11.63
Mobp	12.44	12.08	12.36	12.24	12.69
Wipf1	2.22	1.74	2.02	2.03	1.71
Rat liver dataset					
Bax-AFL	1.78	1.86	1.75	1.96	2.62
Cdkn1a-AFL	4.19	4.28	3.30	8.00	23.50
Myc-AFL	0.97	1.03	0.82	0.99	2.10
Met-AFL	−1.02	−0.94	−0.88	−0.89	−1.90
Bax-NIT	1.62	1.22	1.19	1.25	1.98
Cdkn1a-NIT	3.22	2.82	2.56	3.05	8.07
Figf-NIT	4.18	3.76	3.51	3.72	10.27
Fzd4-NIT	−0.30	−0.70	−0.69	−0.73	−2.07

It is important to note that depending on the tool used to perform differential expression analysis and the dataset it was implemented on the correlation between RNA-seq and qRT-PCR expression estimates varied significantly. In particular, we observed an equally good performance of Cuffdiff, edgeR and DESeq2 on the mouse cortex dataset, whereas in case of human hippocampus and rat liver datasets Cuffdiff performed better than any of the R packages. One possible explanation is that since Cuffdiff utilizes an approach to model count distribution that is conceptually different from of edgeR and DESeq2, it might perform better at modeling read distribution in all three datasets, whereas edgeR and DESeq2 does not model rat and human datasets as adequately.

Therefore, CANEapp demonstrates excellent performance in estimating gene expression changes in a variety of experimental designs and using RNA-seq data produced with different experimental and sequencing protocols, as well as alternative analysis approaches. This indicates that CANEapp is not only user-friendly and adaptable to different computational platforms, but it is also a robust and accurate tool to perform differential gene expression analysis. Moreover, the ability to perform analysis of differential gene expression with alternative tools in parallel makes CANEapp useful for performing benchmarking experiments.

### Discovery of novel long noncoding RNAs using CANEapp and their experimental validation

**Fig. 4:**
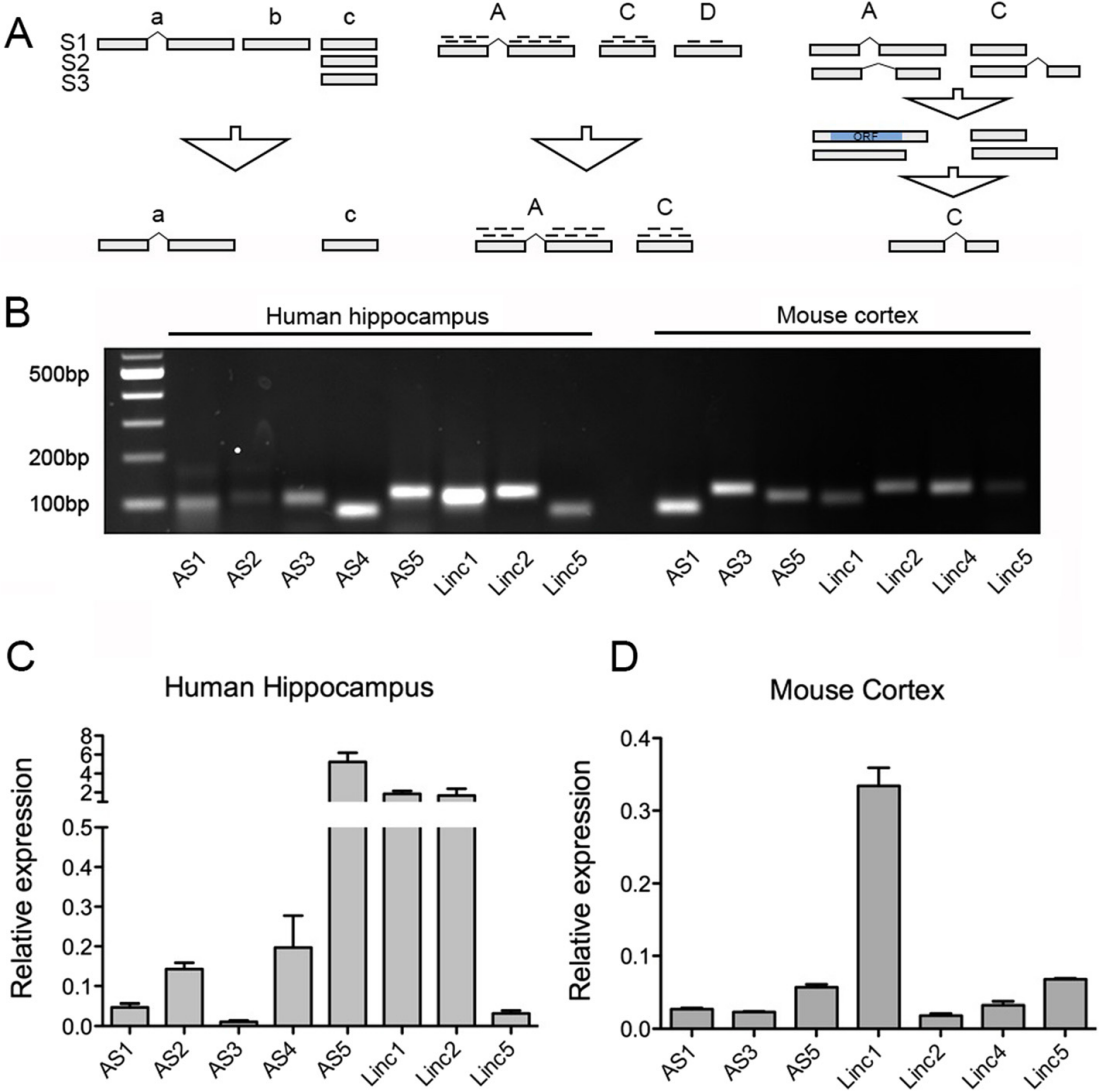
Detection and of novel long noncoding RNAs by CANEapp and their validation by real-time PCR. **a** Filtering strategies and protein-coding potential prediction. (Right) CANEapp preserves any transcripts that contain a splice junction (**a**) or single-exon transcripts expressed in a majority of samples (**c**), whereas single-exon transcripts detected in a minority of samples are filtered out (**b**). (Center) Loci that have insufficient read coverage are not considered for differential expression testing. (Left) In order to differentiate between novel noncoding RNAs and potential protein-coding genes, each isoform from a novel locus is tested for presence of a significant open reading frame. Loci that contain at least one isoform with an open reading frame are not considered novel noncoding RNA. **b** Gel electrophoresis image of PCR amplification products for experimentally validated novel long noncoding RNAs**.** 5 novel antisense RNAs and 3 long intergenic noncoding RNAs (lincRNAs) predicted from the human RNA-seq dataset analysis were amplified with real-time PCR. For mouse cortex dataset, real-time PCR was performed on RNA extracted from adult mouse cortex. 3 antisense RNAs and 5 lincRNAs were successfully validated. **c** and **d** Novel long noncoding RNAs span a wide range of expression levels in human and mouse tissues. Relative expression of validated long noncoding RNAs was calculated by normalizing it to the Ct value of the endogenous control beta-actin

It is becoming more and more evident that the ability to extend analysis of transcriptomes beyond expression changes in annotated gene loci and transcripts is indispensable to elucidating normal cellular processes and pathological states [26–30]. For instance, a recent study analyzing thousands of RNA-seq datasets from normal tissues and cancers have annotated ~50,000 novel long noncoding RNA transcripts and have implicated these transcripts as important markers of cancer subtypes and normal tissues types [31]. Therefore, a true cutting-edge RNA-seq analysis package must include the functionality to perform accurate discovery of novel transcripts. CANEapp peroforms *ab initio* assembly of transcripts that is not dependent on previous transcriptome annotations and allows discovery of unannotated transcripts [4, 15]. It includes a workflow (Fig. 4a) that filters single-exon transcripts that potentially originate from transcriptional noise or sequencing artifacts, filters out lowly expressed loci and classifies novel loci into noncoding RNA or potential novel protein-coding genes.

In order to experimentally validate the expression of predicted novel long noncoding RNAs from the human and mouse datasets, we used CANEapp’s primer design feature to design exon-junction spanning primers (Table 5) and performed RT-qPCR experiments on RNA extracted either from human hippocampus or mouse cortex. Expression validation of novel lncRNAs identified in mouse cortex and human hippocampus was performed by SYBR Green-based qRT-PCR analysis. 1 μg of mouse cortex and human hippocampus RNA were converted to cDNA using the high capacity cDNA synthesis kit from Life Technologies. 1 μl of diluted cDNA was used for SYBR Green-based real time PCR analysis. Expression of each gene was normalized to Ct value of beta actin. Amplification specificity was assessed by the presence of a single peak in the melting curve analysis and by checking the size of the amplified products on 2 % agarose gel electrophoresis.

**Table 5:** Primer sequences for validation of novel noncoding RNAs

Human Hippocampus	
AS1	Left Primer: ACTGGAGAAGCACGGGGA
	Right Primer: AAGTTCCACGTGGCTGGG
AS2	Left Primer: TCGAGCTGAGGACGTGGA
	Right Primer: TTTCCTGCCTGGCTGGTG
AS3	Left Primer: TCCCTGTGTGTCTGCACC
	Right Primer: CCCACACTCAGTTCTTCCCA
AS4	Left Primer: AGAGCGGTAGGGATACGCT
	Right Primer: GCTGCTGATGGGTGGTCC
AS5	Left Primer: CCATGCCTAGCCTCAGGG
	Right Primer: CTATGTGAGCTTGGGCAAGT
Linc1	Left Primer: CTGCCCTGTGGAGCATCC
	Right Primer: CTCTGGCAAGGCGTTCCA
Linc2	Left Primer: CCTGGCACCGCAGCAA
	Right Primer: GCTGTCCTAATGCTTCATCCA
Linc5	Left Primer: CAGGGCCCAGGATCCAGA
	Right Primer: TGAATTACTGCCACGACCAAG
Mouse Cortex	
AS1	Left Primer: GCCCAGGCTCTCCAGAGA
	Right Primer: ATAGTCCCTCTCCCCGCC
AS3	Left Primer: ACGAAAGGGTGCCTTCCC
	Right Primer: GCTTACTCCCGTCACCCC
AS5	Left Primer: TTCTTGGACAGCGACCCC
	Right Primer: AGCGTCAGGAAATGGCCA
Linc1	Left Primer: TCAGGAGAAGCAGCGTGC
	Right Primer: TCCTTCTCCAGATCTCAGGGT
Linc2	Left Primer: TGGTCATGAACTTGTTCCTGT
	Right Primer: GCCTGGACTCCTATGCTCA
Linc4	Left Primer: CCAGGAACGGCTGAGACG
	Right Primer: CTCACAGGCCAGCTGGAG
Linc5	Left Primer: GCTGCTCCGAGCTCAGTC
	Right Primer: TTTGGAGCGGTCCTGCAG

Overall we designed primers for 20 novel long noncoding RNAs, 10 for each dataset. 10 of those were antisense RNAs and 10 were long intergenic noncoding RNAs. We could accurately detect expression of 15 (75 %) out of 20 predicted transcripts, as is evident from the gel electrophoresis image of real-time PCR reaction products (Fig. 4b). Therefore, our novel RNA prediction workflow and primer design software were accurate and robust in two different datasets; since we used only one primer set per transcript, using a second set of primers would probably increase the rate of successfully detected transcripts. Novel long noncoding RNAs identified with CANEapp span a wide range of expression levels (Fig. 4c), suggesting that the software is accurate in detecting both lowly and highly expressed transcripts.

## Discussion

We have developed CANEapp, the first fully automated RNA-seq analysis tool that combines user-friendly graphical interface with the ability to utilize the computational power of high-performance servers. CANEapp can be run from Windows or Mac machines and it automatically connects to the server to transfer raw data and install the components of the analysis pipeline. Thus, CANEapp does not require use of a terminal to communicate with the server and can be used by simply downloading and unpacking the point and click interface. CANEapp includes an intuitive step-by-step experiment design suite and allows seamless monitoring of multiple projects analyzed on different servers. In addition, it stores all the information associated with individual projects, allowing immediate access to previous analysis settings. Due to these unique features CANEapp is immediately accessible for any user with no bioinformatics or computer science expertise and thus mitigates the need of involving bioinformatics experts in analysis of RNA-seq experiments. RNA-seq analysis can be particularly challenging in case of analyzing multiple groups and samples, and the workflow requires optimization and experimental validation to perform robustly in varying experimental and technical conditions. In addition to providing a user-friendly suite to design and monitor RNA-seq analysis projects, CANEapp includes a fully automated, robust and experimentally tested computational pipeline to perform differential gene expression and novel noncoding RNA analysis. The pipeline contains alternative analysis tools for read alignment (TopHat and STAR) and differential gene expression testing (Cuffdiff, edgeR and DESeq2), allowing the user to perform a customizable analysis or even to compare performance of several tools in a single run of CANEapp. We have tested the performance of the pipeline by analyzing three RNA-seq datasets from three species: human, mouse and rat. The test datasets consisted of RNA-seq experiments with different technical sample preparation and sequencing protocols: polyA enriched versus ribodepleted RNA, directional versus unidirectional library preparation, single versus paired-end sequencing. CANEapp has produced extremely accurate estimates of differential gene expression in all three datasets as was validated independently with qRT-PCR. This indicates robustness of CANEapp performance with varying experimental designs and technical sample preparation protocols. In addition, we noticed that Cuffdiff performed equally well on all the three datasets, whereas edgeR and DESeq2 performed worse on the rat and human datasets when compared to qRT-PCR. This result is in line with the observations in the paper describing Cuffdiff2 tool [5] and is contrary to what has been reported elsewhere [32]. Even though comparing performances of different differential expression analysis tools is outside the scope of this paper, we would like to point out a potential use of CANEapp as an easy way to compare performance of Cuffdiff, edgeR and DESeq2 as it allows to implement these tools simultaneously in a single run.

With the evolution of sequencing technologies and bioinformatics tools to analyze RNA-seq data more and more long noncoding RNAs are being discovered. The number of known lncRNAs only in human has exceeded that of protein-coding genes and is still growing [31, 33, 34], potentially because of high tissue and dynamic specificity of these transcripts [35–37]. Therefore, it is crucial to be able to perform discovery of novel noncoding RNAs in order to gain a comprehensive view of the transcriptome of a particular tissue or cell type. CANEapp includes a computational workflow to accurately assemble and predict novel, previously unannotated noncoding RNAs. We were able to experimentally validate expression of 15/20 (75 %) of the novel noncoding RNAs in human and mouse tissues, which correlates with previous reports on accuracy of prediction of novel spliced junctions based on RNA-seq and indicates that CANEapp is a robust and valuable tool for novel noncoding RNA discovery.

Several software packages aiming to provide a user-friendly means to analyze RNA-seq data have been designed. We performed a comprehensive comparison of CANEapp to six other packages for RNA-seq analysis and analyzed several key features of these tools. We demonstrate that CANEapp significantly improves on previously developed tools in a number of ways. For instance, Myrna [38] is a cloud-based pipeline that performs differential gene expression analysis of RNA-seq data. Myrna has an advantage of performing automated analysis in one step; however, among its drawbacks are cumbersome installation, lack of user interface and limited functionality. Since Myrna relies on an ungapped aligner it can only perform analysis of annotated genes and is not able to analyze splicing events or novel RNA species. The Galaxy Project [39] is an open-source platform with a web interface allowing a user to perform analysis of next-generation sequencing data on a local cluster or Amazon Cloud. However, even though Galaxy simplifies use of bioinformatics tools by providing a web interface and removing the installation step, it still lacks automation and requires a step-by-step analysis of each individual sample; instead assuming that the end user has a deep knowledge of the tools used and is able to construct analysis pipelines and select tools and settings appropriate for individual experiments. RobiNA package [40] is a Java-based tool that performs step-by-step analysis of RNA-seq data to discover differentially expressed genes. However, RobiNA is missing several important functionalities. It works on the local machine, which means RobiNA ‘s performance is severely restricted to the low performance of personal computers and it is unable to operate on servers, engage high-performance computing resources or cloud computing, all of which are the most commonly used platforms in academia to analyze next-generation sequencing data. Moreover, RobiNA uses ungapped aligned Bowtie to align sequencing reads, which means it is not capable of *ab initio* analysis of RNA-seq data to discover novel spliced junctions, transcripts and loci. Other packages such as RNA CoMPASS [41] include additional functionalities such as gapped alignment and ability to work on a server but still require cumbersome installation and configuration that makes it inaccessible to users without computer science skills. In addition, RNA CoMPASS does not include a complete analysis pipeline and is missing many important analysis steps such as differential gene expression analysis, transcript annotation and long noncoding RNA discovery. We believe that CANEapp improves on previously designed packages in many aspects and presents a software that combines user-friendly interface, automation of the analysis, an optimized and experimentally validated analysis pipeline, and ability to perform computation on high-performance and cloud servers. CANEapp is a truly user-friendly powerful tool that can be used without any bioinformatics or computer science expertise to perform comprehensive transcriptome analysis.

## Conclusion

CANEapp potentially represents a novel platform for integrating next-generation sequencing analysis pipelines and tools into a user-friendly suite that can be immediately accessed by scientists. One of the main challenges of high-throughput biology is integrating data from different sources and experiments. CANEapp utilizes a standardized analysis pipeline and internally generated experimental design templates and can be run on any Linux architecture by a non-expert user. The use of a standardized pipeline together with a pre-defined software-generated design template that will include all specification of the biological experiment and technical protocols can serve as a primer to develop a standard way to analyze next-generation sequencing data and high-throughput data in general. This will create an opportunity to integrate data into global databases for sharing and meta analyses. We believe that CANEapp will not only benefit biologists in performing their RNA-seq experiments, but will also inspire and provide bioinformaticians with code source material to develop user-friendly analysis tools for various applications in genomics analyses such as analysis of gene fusions, RNA editing, circular RNA analysis and simultaneous analysis of the genome and transcriptome.

## Ethical approval

There was no ethical approval needed for this study, because it was based on publicly available data sets with de-identified samples.

## Availablity and requirements

**• Project name:** CANEapp

**•Project home page:**http://psychiatry.med.miami.edu/research/laboratory-of-translational-rna-genomics/CANE-app

**•Source code:**https://github.com/DmitryVel/CANEapp

**• Operating system(s):** Windows and Mac (GUI), Linux (serve-side pipeline)

**• Programming language:** Python, Java

**• Other requirements:** Java 7 or higher, Python 2.6 or higher

**• License:** GNU GPL 2.0

**• Any restrictions to use by non-academics:** no

## Abbreviations

NGS: Next generation sequencing
GUI: Graphical User Interface
RNA-seq: RNA-sequencing
qRT-PCR: Quantitative real-time PCR
JSch: Java Secure Channel
SRA: Short Sequence Archive
CNCI: Coding-NonCoding Index
GTF: Gene Transfer File
GLM: Generalized Linear Model
CANEapp: Application for Comprehensive automated Analysis of Next-generation sequencing Experiments
